# Large Scale Environmental Monitoring through Integration of Sensor and Mesh Networks

**DOI:** 10.3390/s8117493

**Published:** 2008-11-24

**Authors:** Raja Jurdak, Abdelhamid Nafaa, Alessio Barbirato

**Affiliations:** 1 CSIRO ICT Centre, QCAT Technology Court, Pullenvale, QLD 4069, Australia; 2 Computer Science and Informatics, University College Dublin, Belfield, Dublin 4, Ireland. E-mail: alessiobarbirato@gmail.com

**Keywords:** scalable, mesh, network management, autonomous, wireless, sensors

## Abstract

Monitoring outdoor environments through networks of wireless sensors has received interest for collecting physical and chemical samples at high spatial and temporal scales. A central challenge to environmental monitoring applications of sensor networks is the short communication range of the sensor nodes, which increases the complexity and cost of monitoring commodities that are located in geographically spread areas. To address this issue, we propose a new communication architecture that integrates sensor networks with medium range wireless mesh networks, and provides users with an advanced web portal for managing sensed information in an integrated manner. Our architecture adopts a holistic approach targeted at improving the user experience by optimizing the system performance for handling data that originates at the sensors, traverses the mesh network, and resides at the server for user consumption. This holistic approach enables users to set high level policies that can adapt the resolution of information collected at the sensors, set the preferred performance targets for their application, and run a wide range of queries and analysis on both real-time and historical data. All system components and processes will be described in this paper.

## 1. Introduction

Sensor networks have emerged as an enabling technology for collecting digital samples from physical spaces, as a result of recent advances in microprocessors, wireless communication, and miniaturization. The sensors can be deployed in ad hoc fashion in the area of interest, with no requirement for a communication infrastructure. These nodes, which include tiny processors, memory, low-power wireless radios, and physical sensors, can self-organize to form a wireless network. The low power transmissions of the sensor nodes imply that a single node's transmission has a limited range in the order of tens of meters. To overcome the limited range issue, nodes closer to the central collection node can relay the sensor information of nodes that are further away through multiple hops, until the information reaches a base station that is connected to the Internet. The base station serves as a gateway node for streaming sensor data to the users.

Researchers predict that tiny sensor nodes will be deployed all over our physical world for terrestrial and marine monitoring. However, simply covering large areas with tiny sensors using is not feasible given the extremely limited communication range of these sensors.

We illustrate the challenges of large scale sensor networks through the example of precision agriculture [1], where sensors are deployed in agricultural fields to collect data on soil moisture, temperature, and light exposure of crops. Current sensor architectures rely on a base station node in each cluster to relay data from the deployed sensors to the user through a long haul or Internet connection. Because farmers typically own several fields across a geographic region, they must install a set of sensors (or sensor clusters) in each field. Connecting each sensor cluster directly to the Internet requires as many Internet subscriptions and physical connections as there are clusters, which incurs significant cost and limits the scalability for large-scale agricultural deployments. Another inhibiting factor with legacy systems resides in the fact that managing geographically-distant sites is done through completely different interfaces, where the user has no integrated view of the sites, which hampers the data analysis and decision-taking processes.

A second challenge arises from static time-driven monitoring each commodity individually, which provides users with highly detailed information, but it often represents an overkill for their application needs. Synthesizing measurement information can improve the user's interpretation of the data and thus the efficiency of the decision-making process. For example, providing temperature information from each sensor within the same field is highly redundant. This causes a large energy overhead that speeds up battery depletion at the sensor nodes, and increases the cost of frequently replacing sensor batteries. Although energy efficient techniques, such as data aggregation, exist, their configuration currently requires technical know-how that is not accessible to the system end-user.

Finally, using the service provider network for cluster-to-cluster and cluster-to-user communication also limits the end-user's ownership of the information paths, which may represent a security risk, as well as a cause of added cost. In current architectures, any cooperative exchanges between clusters must traverse the service provider network, which could allow a competitor or a malicious third party to intercept this information.

**Figure 1.**
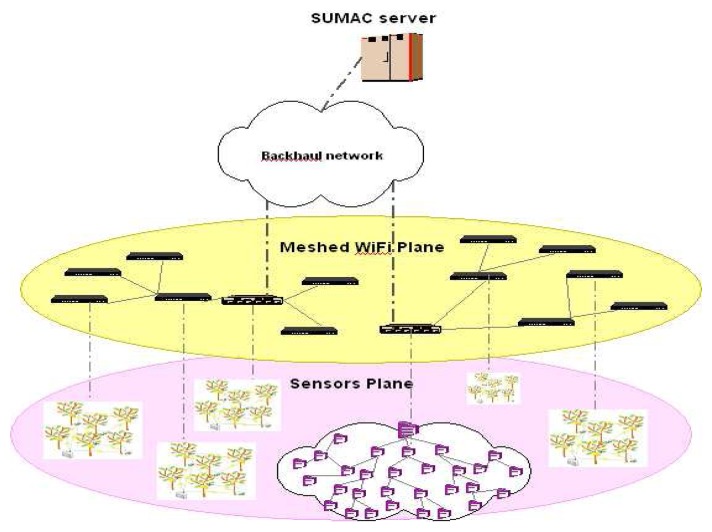
The SUMAC Architecture.

To address these challenges, our project on Scalable and Unified Management And Control of geographically dispersed sensors (SUMAC) aims at enabling unified monitoring multiple dispersed physical areas, through an architecture which includes a medium range wireless mesh network that serves as a bridge between geographically-spread sensor node clusters and the Internet, as shown in Figure 1. The project involves the design of an integrated communication protocol suite within the architecture to reduce the required Internet subscriptions in order to provide users with full ownership of data communicated within their network, that is easily manageable, secure, fast, energy-efficient and inexpensive.

This paper provides an overview of the SUMAC architecture and its main components, including the sensors plane, the mesh plane, and the server plane. The paper presents the sensor-related optimizations of SUMAC, including: (1) a unicast reverse routing (downstream) strategy, which builds on a distributed and unique addressing strategy, for avoiding broadcast dissemination, (2) a versatile user-configurable cost function that includes energy, delay, and reliability metrics, and (3) an adaptive fidelity feature, which enables network users to set the data resolution level based on simple high level performance policies. While the mesh network serves as a communication bridge between the sensors and the server, the SUMAC server architecture supports historical data collection from large scale deployments, real time data access, as well as high flexibility in configuring the sensors. The paper also provides empirical results from our lab testbed that explore the energy/delay tradeoff of the adaptive fidelity mechanism, and how this tradeoff can translate to autonomous delivery of user performance requirements.

SUMAC provides end-to-end scalable methods that run across the three network planes for user configuration of the sensor node performance settings. These methods enable the setting of high level policies and performance targets that dynamically adapt the fidelity (resolution) of the incoming sensor data stream and the routing strategy of packets within the sensor network to events of interest in the deployment region. The high level settings by the user trigger a background process that sets the default data aggregation level (data resolution) and that determines conditions and rules for overriding or modifying the default aggregation level autonomously by the network. For instance, the default aggregation level could be set to average most of the sensor data in a single cluster, which saves energy. When an event of interest occurs, the sensors can automatically slide down the aggregation level to enable nodes to send their raw packet data in order to monitor this event's evolution more closely with higher data resolution. The background process also sets the routing cost function weights in the sensor clusters, which determines routing paths in the network and can be tailored to favour system lifetime, data quality, or high responsiveness. This process also supports a feedback mechanism, by which a change in network state resulting from a user configuration change is continuously reassessed at the server, and appropriate reconfiguration adjustments are done accordingly and autonomously by the server. While the user could set up custom alerts for getting notification of events of interest, all reconfiguration of network metrics occurs transparently according to the high level policies set by the user.

The remainder of the paper is structured as follows. Section 2. positions the paper with respect to previous work on large scale sensor architectures, emerging commercial solutions, and aggregation techniques. Section 3. describes the SUMAC architecture in detail, introduces its components, and discusses each component's design features. Section 4. presents our empirical results for adaptive fidelity sensing, while section 5. discusses the results, future work, and concludes the paper.

## 2. Related Work

Many research groups and commercial companies are considering the interoperability between wireless sensor networks and other network technologies as the viable solution for the large-scale sensing coverage. The idea of using a hierarchical architecture to achieve this purpose is not novel in and of itself, but to the best of our knowledge, a comprehensive large scale monitoring solution that is accessible to non-specialized users has yet to be provided. Intel's project on “Heterogeneous Sensor Networks” [5] adopts an approach similar to SUMAC's. It uses a two-tier wireless network, including an 802.11 mesh network comprised of high-end nodes that overlays a sensor network. This project's use of a similar architecture to SUMAC confirms the commercial relevance of this approach for real world monitoring applications. The focus of Intel's project is on the performance improvements, in terms of energy and delay, resulting from the use of fixed power supply devices, such as 802.11 mesh nodes. While SUMAC also focuses on tradeoffs among energy, delay, and data resolution, the focus of our project lies more on the software side, and in particular, on securing an end-to-end data path that includes intuitive user access, control, and customization of the network in a unified manner. In addition, the SUMAC architecture targets applications where power supplies are only available at the cluster edges, such as in outdoor monitoring applications.

Another interesting work is the one proposed in the ART-WiSe networks [6] that provides an overview of the architectural requirements and behavior of a gateway capable of managing two different interfaces: IEEE 802.15.4/ZigBee and IEEE 802.11e. They focus their analysis on the QoS provision. The authors of [7] explore the potential application of a decentralized two-tiered architecture in large scale wireless sensor networks where the second tier is the basic WLAN that uses the IEEE 802.11 protocol. They observed that wireless sensor networks with a solid backbone like the one offered by WLAN's, can better handle issues like real time performance, reliability, scalability and cost efficiency. The preliminary performance evaluation of the SUMAC components have also confirmed their conclusions.

Several industry-based solutions have also started addressing the need for large scale sensing through heterogeneous networks as well. Existing solutions that promote integrated large scale network architectures include both hardware platforms and software packages. Hardware platforms, such as Meshnetics ZigBit Ethernet Gateway (www.meshnetics.com), Arch Rock Premier Pack (www.archrock.com) and Digi Connect Port X (www.digi.com) enable communication between different networks, but simply integrating multiple wireless interfaces into the same box can cause congestion, delay and loss of important data in certain regions of the network, because they do not support transparency of state information between networks. Our work aims to develop software mechanisms that leverage existing multi-modal hardware and that provide quicker and more reliable network bridging, avoiding the creation of congestion hot spots in the network. In particular, our user-configurable cost function supports sharing of state information between the WiFi network and the sensor clusters (which use IEEE 802.15.4 technology), to enable the sensor nodes to choose the best WiFi gateway to use, based on the congestion level at potential gateways and on delay, battery level, link quality, and hop count metrics of the forwarding path from the node to the backhaul. As a result, nodes may sometimes choose a WiFi gateway that is not necessarily the nearest physical gateway in order to avoid creating congestion hotspots in the WiFi network, or to spare low-battery sensor nodes, increasing their lifetime.

Other existing solutions for integrated large scale network architectures provide software packages for streamlining data delivery in the network. ArchRock (www.archrock.com) provides one such solution that relies on Internet Protocol addressing, in an effort to bring IP technology to every sensor device, so that any commodity is addressable through the Internet. This work, referred to as 6LoWPAN [11], relies on address translation at the sensor cluster gateway in order to use short 2-byte IP addresses for the sensors to avoid the overhead of full size IP addresses. While using short IP addresses for each sensor node is an attractive notion, it involves the drawback that dynamically assigning these addresses for a large number of sensor nodes through a centralized Dynamic Host Communication Protocol (DHCP) involves delay and communication overhead. A alternative distributed DHCP implementation risks address overlaps, as two WiFi gateways that are in different regions of the network may assign the same address to two different nodes, which causes confusion when the user tries to access one of the nodes. Assigning IP addresses statically would avoid the drawback of dynamic assignment, but it is not flexible enough to accommodate varying cluster memberships. SUMAC avoids using a centralized DHCP server, by using IEEE 802.x MAC addresses for identification of nodes both within the WiFi mesh network and the sensor clusters. This addressing strategy ensures unique node addresses, as IEEE 802.x address are hard-coded into each device. It also provides higher flexibility over static IP address assignment, as nodes can move around different clusters while keeping their addresses, and it avoids the delay and communication overhead of assigning addresses dynamically through DHCP and of resolving address conflicts.

Other software solutions for sensor networks are openly available. The current state-of-the-art in sensor networks provides extensive open-source network management mechanisms and protocols at the cluster level, using operating systems such as TinyOS [8] and Contiki [9]. However, the management of *multiple* sensor clusters in an integrated way, which is the focus of the SUMAC project, has not yet been addressed within these open-source systems. Global Sensor Network (GSN) [10] provides an open-source middleware java environment that runs on a back-end server of a sensor network. GSN supports real and virtual sensors, enabling the abstraction and integration of any type of sensor. While the SUMAC server architecture resembles GSN in some aspects, SUMAC's server design aims at supporting large scale deployment of many disjoint sensor clusters that converge to wireless mesh networks. The higher data volume involves more complex data management strategies at the server that go beyond relational database concepts.

One particular optimization technique for large sensor networks is in-network aggregation, which is crucial for WSN's where energy resources are limited. Widmer et al. [2] present a comprehensive review of techniques and protocols for in-network aggregation in wireless sensor networks. There is evidently no aggregation standard, but aggregation strategies can be differentiated depending on features like timing and function. Widmer et al. [2] identify three main timing strategies: (1) periodic simple aggregation, (2) periodic per-hop aggregation, and (3) periodic per-hop adjusted aggregation. While (1) implies that each node has to wait a predefined period of time before aggregating and sending the packet, (2) and (3) add the functionality of sending the message before the firing of the timer at the aggregating node once it has already received all the incoming packets from its children. In (3), the timeout of the node is managed automatically depending on the position of the node in the tree. Our implementation uses a strategy similar to (1) to account for potential instability of network topology. As node paternity varies over time, the node cannot predict the group of motes from which it expect to receive messages. The aggregation functions defined by [2] include information lossy aggregation and information lossless aggregation. Our project targets long term operation, so we have opted for lossy aggregation by simply taking the average, maximum, or minimum value.

Directed diffusion [3] is a simple periodic data collection protocol for sensor networks that targets the monitoring of events which are typically sensed only by a few nodes. In this algorithm, the base station broadcasts requests for relevant data and according with the nodes answering, it waits for the information response. When the node responds it automatically generates a path. A node that belongs to different data paths is in the right position to aggregate data coming from different sources.

A similar approach is Greedy aggregation [4]. Greedy aggregation differs from Directed aggregation in path establishment and maintenance. Directed diffusion establishes paths using path reinforcement; a node may locally decide to draw data from one or more neighbors in preference to other neighbors. In directed diffusion, exploratory samples have initially and repeatedly been flooded throughout the network. In the Greedy approach, in addition to the normal information (delay), each exploratory sample also contains an energy cost for delivering this sample from the source to the current node. Each source on the existing tree also generates an on-tree incremental cost message which corresponds to each new exploratory sample received. The incremental cost message contains the incremental energy cost required for delivering the corresponding exploratory sample to the existing tree. In this Greedy approach, the most preferred neighbor is the one that has delivered one of the two messages at the lowest energy cost.

Our aggregation approach also considers energy costs for path generation, albeit in a slightly modified way. Nodes share their energy consumption and buffer size values with immediate neighbors. This local neighborhood information, in addition to path Etx and delay, drive routing decisions. Furthermore, our aggregation mechanism is coupled with a powerful yet simple user interface for visually setting aggregation rules on the basis of several conditions: hop count, resolution, sensor values, sensor tags, GPS coordinates.

## 3. The SUMAC architecture

### 3.1. Overview

The SUMAC architecture introduces an intermediate network plane, in the form of a wireless mesh network of IEEE 802.11 (WiFi) nodes, which serves as a bridge between geographically spread sensor clusters and the service provider network, as illustrated in Figure 1. The proposed network architecture entails three network planes:

1. Geographically disjoint 802.15.4 [12] sensor clusters that are deployed to monitor the user commodities locally. Each sensor cluster forms an independent wireless sensor network that delivers collected data to a gateway node.
2. An 802.11 [14] WiFi meshed network that relays sensor data to the backhaul network. Because WiFi technology provides a larger communication range than wireless sensor nodes, the WiFi mesh network serves as a multi-hop access network for the sensor clusters, acting as a bridge between the sensors and the backhaul. Certain multi-modal nodes that are equipped with both IEEE 802.15.4 and WiFi technology serve as a gateway for a sensor cluster.
3. A remote central server that acts as a backend for a web portal that delivers added-value visualiza tion, control, storage, and analysis features to the user.

The integration of the above network planes reduces the number of required Internet subscriptions for a given set of clusters, as the mesh network can relay sensor cluster information to the nearest Internet-connected node. The one-time deployment cost of WiFi mesh nodes is outweighed by the cost reduction of using fewer Internet subscriptions. The WiFi mesh overlay also creates an opportunity for reducing security risks and latency for communication between neighboring sensor clusters. The SUMAC architecture consolidates these three planes for seamless coordination and information delivery to users.

The remainder of this section discusses each plane, including its design, challenges, and current development stage, separately.

### 3.2. Sensors Plane

The sensors plane in SUMAC is responsible for collecting data from physical spaces and relaying this data towards a central base station in a multi-hop fashion. All nodes that report to the same base station constitute a sensor cluster. Each cluster of sensors monitors a contained physical space, such as a building or an agricultural field. The SUMAC architecture supports networks that are deployed in ad hoc fashion or on the basis of predetermined locations.

A central objective of the SUMAC project is to capitalize on existing developments for intra-cluster sensor communication and to extend them for supporting the management of multiple sensor clusters through a unified interface. Achieving this objective demands addressing several challenges, which we discuss below.

#### Collaborative Topology Management

**Challenge:** The first challenge is the transparency of routing state information between the overlay WiFi network and the sensor clusters, which can lead to network congestion. For example, sensor nodes may forward data to a particular WiFi gateway if the gateway seems underused from the clusters perspective. However, the WiFi network may be highly congested at this particular gateway, but current techniques hide this information from the sensor nodes. This problem is further exacerbated in ad hoc deployments where the topologies of the sensor clusters and the mesh network are neither symmetrical nor known before deployment.

**Solution:** To address the issues of transparency between networks and of tunable performance settings, SUMAC proposes a collaborative topology management (CTM) strategy that supports scalable, flexible, and seamless two-way access to the sensors. CTM relies on transmission of periodic beacon messages that nodes use to notify their direct neighbors about their current conditions. The CTM strat egy include an additive cost function in the beacons to compute the routing cost for each neighboring node, as follows:

(1) $${\mathrm{Cost}}_{\text{CTM}}(N)=\alpha \text{ETX}(N)+\beta E(N)+\gamma D(N)$$

where *ETX* is the Expected number of Transmissions metric, *E* is the energy consumption at the send ing node *N*, *D* is the delay measured by the buffer length at node *N*, and *α*, *β*, and *γ* are the user-configurable cost function weights. *ETX* is a standard routing metric in sensor networks that combines information on link quality and the number of hops from the base station. The energy and delay metrics have been added in the periodic beacon messages structure as well as in the routing table. Including these two metrics in the weighted cost function enables the use of high level policies to change the low-level logic that selects a routing parent considering these new parameters. Once a node receives and stores its neighbors' beacons, a dedicated timer at each node fires periodically and launches a task that selects the lowest cost neighbor in the neighbor table.

Users can set the cost function weights, *α*,*β* and *γ* through the graphical triangle tool in the web portal, which is discussed further in section 3.4.. Additionally, the user can adjust higher level policies to set up relative priority of metrics, which the server translates into cost function weight assignments that are disseminated to the node, for e.g. energy consumption versus lower delay and high throughput.

The cost function also provides visibility of network state information across gateways, so that a sensor node can dynamically determine the optimal gateway to use based on a more comprehensive set of network performance metrics. This CTM scheme exploits the concept of hierarchical beacon propagation from the WiFi mesh network to the sensor nodes. A sensor node has access to performance metrics regarding the end-to-end path: from its direct sensor parent to the backhaul connection. Our initial tests reveal this CTM strategy will significantly improve network throughput by using less congested paths to the service provider network, and it will reduce queuing delay at the gateways through the selection of the least congested gateway.

#### Surgical Node Reconfiguration

During long deployments, specific nodes may detect events of interest to the network operator. The operator can set high level policies at the beginning of the deployment so that the network can autonomously react to such events without any human intervention. Alternatively, operators can choose to receive alerts for events and to manually reconfigure the affected nodes on a case-by-case basis. Both automatic and manual reconfiguration of specific sensors demand a mechanism that enables the backend server to communicate with one or few sensors selectively, to avoid the energy and communication overhead of flooding the mesh and sensor networks with reconfiguration requests that are meant for only a few sensor nodes.

**Challenges:** Targeted sensor reconfiguration involves two challenges: (1) Unique addressing; and (2) Downstream (reverse) routing.The separate addressing strategies at each network plane in the the integrated network architecture represent a challenge to surgical node reconfiguration. While currently proposed architectures use Internet Protocol (IP) addresses to abstract out the differences in heterogeneous networks [11], IP addresses are assigned dynamically through a central DHCP server, which can cause latency in the address assignment process especially when sensor nodes are mobile, and they are not necessarily unique, which can cause address conflicts, prevent unique access to the sensor nodes, and prompt costly address conflict resolution routines.

**Solutions:** To tackle the addressing issue, SUMAC uses a distributed and unique addressing strategy on the basis of the last 16 bits of each node's IEEE 802.x MAC layer address, rather than conventional network layer techniques. The use of MAC addresses avoids the overhead and complexity of centrally assigning and managing network layer addresses for all clusters, and it ensures that each node within the network is uniquely addressable. As a consequence, network flooding will be avoided when an end-user wants to configure a specific sensor node. The use of 802.x addressing for both the WiFi plane and the sensor plane will reduce address translation delays at gateway nodes, and it favours integration between sensor networks and mesh networks, easing the abovementioned CTM mechanism. Because some sensor node hide their MAC addresses from the operating system, we have also devised a fall-back semi-centralized mechanism for unique and dynamic address assignment.

First, we can ensure that the base station node has an accessible MAC address. The base station, upon starting up, provides its MAC address to the back-end server through the mesh network. The back-end server, which has a comprehensive view of the network membership, checks whether the last 16 bits of the new base station's address conflict with any other node in the network. If so, then the server assigns a unique 16-bit address to the new base station. Upon receiving the new address, the base station then becomes a distributed address server for all nodes in its cluster, and uses the new server-assigned or server-approved address as the base address (BASE) for all addresses within its cluster. When the first node enters the cluster, the base station assigns the node the address BASE+1. The next node to join the cluster gets address BASE+2, and so on. All base stations also store the next address in their non-volatile flash memory, to maintain the state information even in the eventuality of any node resets or crashes. The address assignment strategy at the server considers other existing addresses in the deployment and avoids assigning a new address space that is numerically close to or conflicting with other existing address spaces.

In case even the base station hides its MAC address from the API, the base station requests a BASE address from the Mesh Server. The Mesh Server can then assign a BASE address to this new base station. The address assignment process then proceeds within the cluster as described above.

Leveraging this addressing strategy is a unicast reverse routing mechanism that enable unicast transmission of node reconfiguration requests in the downstream direction, i.e. from the base station to the sensors. Conventionally, reconfiguration requests in WSN's are broadcast to all the nodes. Even if the user requires reconfiguration of a single node, the broadcast dissemination strategies flood the network with the request. All nodes receive the packet, check if the request targets them, then rebroadcast the packet. This obviously incurs large transmission and energy overhead. Instead, sensor nodes in the SUMAC architecture snoop on all upstream messages they forward in order to extract the originating node ID of the packet and the last forwarder that sent the packet here. This snooping mechanism enable each node to construct a reverse routing table of all the nodes below it in the logical topology with no communication overhead. Having the reverse routing table available locally, when a node receives a downstream request, it checks the destination ID of the request. If the destination is available in its reverse routing table, the node forwards the packet in *unicast*. Otherwise, the node discards the packet. This reverse routing strategy aims at reducing control overhead of broadcasting requests all over the network. For the user, reverse routing enables surgical reconfiguration of specific sensor nodes with minimal overhead to other nodes.

**Figure 2.**
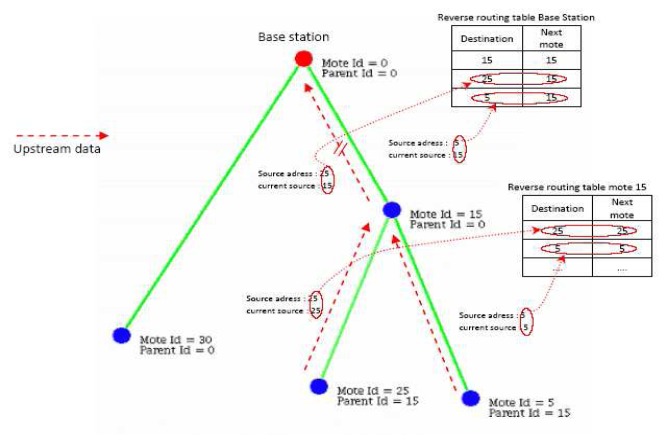
Reverse routing example.

Figure 2 provides an example of the reverse routing strategy. Each packet includes the originator ID and the current sender ID. When node 15 receives packets from nodes 5 and 25, it can add them to its reverse routing table, indicating that these nodes are located in branches downstream of node 15. Since they are direct neighbors of node 15, the next hops to reach nodes 5 and 25 are simply nodes 5 and 25 respectively. At node 0, packets from node 5 arrive with an originator ID of 5, and a sender of 25. Node 0 creates an entry for node 5 in its reverse routing table indicating that node 25 is the next hop to reach node 5 in the downstream sense. Through this strategy, nodes only store next downstream hop information locally, which scales well with large topologies.

#### Adaptive Fidelity

**Challenge:** The final challenge is the sheer traffic flow to and from the sensors, which can cause channel saturation at some nodes and quick depletion of battery energy. As the network includes more sensor nodes, the traffic requirements for accessing and controlling each node individually grows exponentially. This challenge is due to the inflexibility of a static reporting policy, where nodes can only report data or respond to user requests individually.

**Solution:** To provide a more flexible and efficient means to communicate with the sensors, the SUMAC architecture enables the system user to adapt sensing fidelity from the Web portal on the basis of any combination of the following metrics:

- **Hop count:** users set an aggregation level based on a certain hop count *H*. Packets originating at nodes that at least *H* hops away from the nearest base station are aggregated.
- **Data resolution:** users set a data resolution level *DR* indicating how many sensor values should be aggregated. Each node in the network then checks whether it has at least *DR* descendants in the routing tree, and if so, the node does not aggregate its packets. Otherwise, the node aggregates its packet with all other packets awaiting forwarding in its buffer.
- **Sensed values:** users set minimum and maximum thresholds for each sensed value. If the sensed value exceeds this range at some node, the node sets a flag in its packets to override the active aggregation policy. Furthermore, users can set policies that specify whether only the node that detects an exceeded threshold, the set of neighboring nodes around it, or all the nodes in the cluster should override aggregation to deal with this event.
- **Sensor labels:** users can set tags or labels to each sensor node. For example, users can assign a label of *A* to a set of nodes in a vineyard and a label *B* to another set of nodes in a nearby forest. Users can then configure nodes with label *A* to aggregate their packet at a hop count of 5 or more, while setting a data resolution of 10 data points per aggregated value for label *B*.
- **GPS coordinates:** users set aggregation policies on the basis of geographic coordinates of the sensor nodes, by selecting nodes through the Google Maps interface of SUMAC, which is discussed further in Section 3.4.

**Figure 3.**
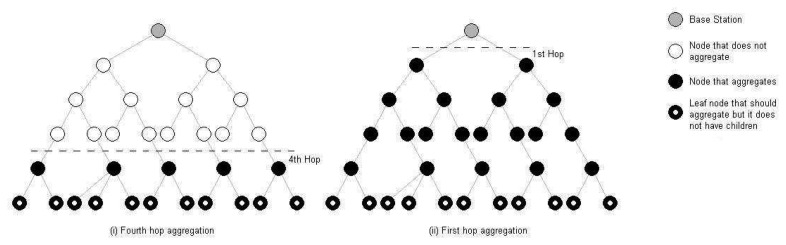
Adaptive fidelity: how it works.

Figure 3 illustrates adaptive fidelity monitoring on the basis of hop count. In this example, the user sets a policy that nodes at one hop or more from the base station should aggregate their data. This effectively means that all nodes in the cluster are aggregating their data when no interesting events occur in the network. The user also determines that in case any node exceeds a temperature of 70 °C, the aggregation level should slide down to hop 4, as this may indicate a fire. Upon detecting that the sensed value exceeds 70 °C, a group of nodes begin overriding the aggregation policy, which in turn causes the base station to broadcast a message to all nodes instructing them that the new aggregation level is hop 4. At this point, each node that is at 4 hops or fewer from the base station stops aggregating its data, while the remainder of nodes continue to aggregate their data.

The idea here is to dynamically slide the aggregation level when nodes detect events of interest, so that the user can have more detailed information about these events, and to return to the original level when measurements return to normal values, which promotes energy efficiency, as our empirical results in Section 4. confirm. These results indicate that the energy savings of adaptive fidelity access and control of the sensors can double the operative lifetime for these battery-operated devices even in small clusters of 20 nodes. Yet another major advantage of the adaptive fidelity resides in the automation of monitoring tasks with reduced user administration; a user may set-up high level alerts and policies, so the network is configured to dynamically re-adjust the resolution of the measurements based on specific events.

#### Auto-reconfiguration through Feedback

**Figure 4.**
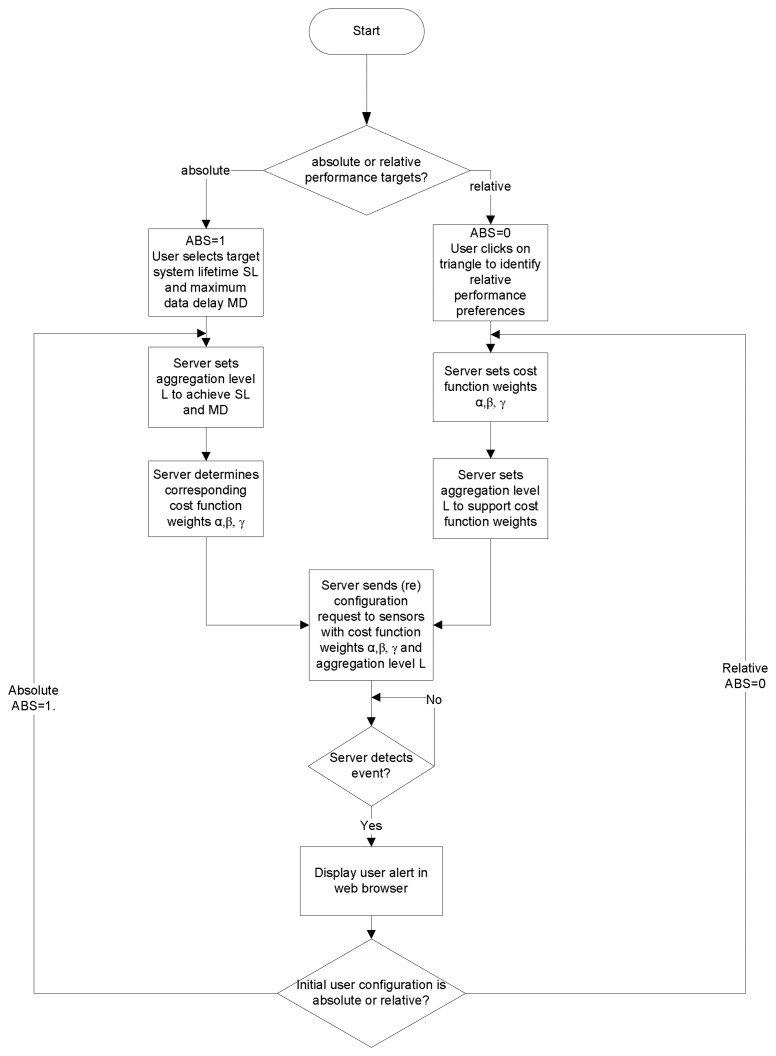
The feedback mechanism at the SUMAC server.

**Figure 5.**
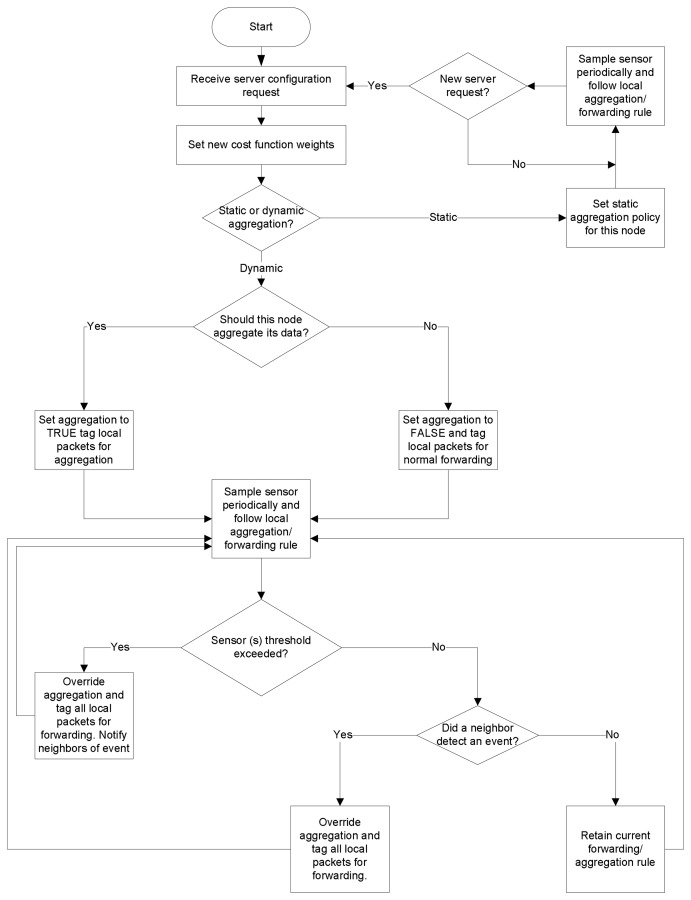
The effect of the feedback mechanism at the sensors.

A final feature of the sensors plane is the feedback mechanism that constantly monitors the state of the network at the server and sends reconfiguration requests to the sensor nodes to maintain adherence to the user-set policy. Figure 4 illustrates this feedback mechanism at the server. Users initially set performance policies in relative or absolute terms. Setting performance policies in relative terms entails setting the relative importance of energy, delay, and quality through the graphical triangle tool shown in Figure 8(a), which we discuss further in Section 3.4.. To set an absolute performance policy, the user can simply select a target battery lifetime or maximum delay for the system through the sliders available in the Web interface. The user also set the sampling period and duty cycle for the sensor nodes. These settings trigger a background analytical process that sets the aggregation (fidelity) level, and the weights for the CTM cost function. The server then sends configuration requests to the selected sensors through the Mesh network plane. Upon receiving the requests, the sensors process these requests and adjust their aggregation rule and cost function weight settings accordingly, as shown in Figure 5.

Note that both relative and absolute performance settings can adapt to dynamic topology changes, including ad hoc deployment of nodes. For relative performance settings, the server remains agnostic to the network topology, as it only needs to set relative metrics. For absolute performance settings, the server considers the current topology of the network, which determines delay and energy consumption for the nodes, by examining the database. Based on the current network topology, the background process at the server determines the new network configuration and informs the sensor nodes.

Sensor nodes distinguish between two types of aggregation rules: static, and dynamic. With static aggregation, the user selects nodes based on GPS coordinates, label, or ID, and sets the selected node to aggregate or to forward their data. In this case, the node aggregation rule is statically set from the server, and the node need only follow this rule until a new server request arrives for this node.

With dynamic aggregation, the user sets thresholds (maximum and minimum) for the sensed values, which in the current implementation are temperature, light, and humidity. If the temperature, humidity, or light value exceeds a user-set threshold at a particular sensor, the sensor node then autonomously decides to stop aggregating the packets and it instructs neighboring nodes to do so as well, in order to monitor the region around this event of interest. This represents region-based aggregation, which users can choose to enable or disabled. If disabled, then only the sensor node that detects the event will override aggregation, while neighbors follow their normal aggregation policy.

The server then notices that some sensors have detected an event of interest, and autonomously reeval-uates the settings for this region in the network. If the user had set absolute performance targets (ABS=1), the server first determines a new aggregation level *L*^1^, and then determines the corresponding cost function weights *α*, *β*, and *γ*. Otherwise, if the user had initially set relative performance targets (ABS=0), the server determines new values for *α*, *β*, and *γ* and then a new aggregation level *L*^1^. The rules for setting the aggregation level based on the cost function weights or vice versa are determined on the basis of empirical experiments that provide rules of thumb for the delay/energy tradeoffs involved. Note that the new aggregation level *L*^1^ instructs the nodes of the new number of hops from the base station beyond which all data packets are aggregated. If *L* > *L*^1^, setting the new aggregation level as *L*^1^ effectively means sliding the aggregation level up, or reducing the data fidelity. In contrast, if *L* < *L*^1^, then setting the new aggregation level as *L*^1^ effectively means sliding the aggregation level down, or increasing the data fidelity. This is what is meant by adapting the aggregation level, or adaptive fidelity.

Once it determines *L*^1^ and *α*, *β*, and *γ* the server then issues a reconfiguration request to the selected sensor nodes with these new parameters. The sensor nodes receive the reconfiguration request and adapt their settings accordingly. When the sensor nodes in the region of the event of interest no longer detect any sensor values that exceed the threshold, they return to their default aggregation/forwarding rule, as initially set by the user. This return to the default configuration is done automatically, as the sensors check whether their sensed value exceeds the threshold periodically, in particular, every time they sample the sensor values. At the first instant that a node detects that the sensed values have returned to normal, it reverts to its original configuration almost immediately.

### 3.3. Mesh Plane

The mesh network plane serves as a medium range overlay that extends the coverage region of the sensor clusters and enables data convergence from multiple clusters towards a single back-end server. Gateway mesh nodes, which interface the mesh and sensors plane, can be deployed uniformly or randomly, depending on application requirements. For instance, outdoor monitoring applications provide limited, if any, access to outlet power supply for mesh nodes, which in turn constrains the placement of the gateway nodes.

There is currently a lot of ongoing activity on providing quality-of-service guarantees within mesh networks, such as in the EU CARMEN project [13]. The SUMAC project complements these and is concerned with the communication at the edges of the mesh network, in particular the interaction of the mesh network with the sensor clusters, and with the back-end server. SUMAC currently uses MikroTik RB600 router boards (www.mikrotik.com), which incorporate a PowerPC architecture and supports up to 3 802.11 a/b/g interfaces. SUMAC relies on gateway nodes that integrate a sensor mote (the Tmote Sky [15]) with a mesh box through a Tmote Connect device [15] that provides USB to Ethernet conversion and runs a serial forwarder).

When a mesh box is connected to a TMoteConnect device, we refer to it as a Clusterhead, which means than a java daemon is launched, called ClusterheadForwarder, and this daemon is responsible for connecting the serial forwarder on the TmoteConnect and grouping incoming sensor packets into larger UDP packets for forwarding to the server.

As the mesh plane is the bridge between the sensor plane and the server plane, we have verified the full two-way data communication path starting from the sensors, through the base station, traversing the mesh network, and reaching the server. Currently, we are performing stress simulation tests on the buffering and forwarding mechanisms at the Clusterhead with thousands of sensors.

### 3.4. Server Plane

The SUMAC server plane is the convergence point of the incoming data from all clusters of a single deployment. Each group of mesh network nodes report their data to a Mesh Portal. Mesh Portals in turn report the data to a single Mesh Server, that represents the bridge between the Mesh Plane and the Server Plane. The connection between the Mesh Server and Mesh Portal uses Secure Socket Layer (SSL), as it usually traverses third party long haul networks or the Internet. The Mesh Server receives streaming data from the Mesh Portals and stores it in a temporary table for provision to the client Web browser. It also forwards the packets to the database for long term storage through the DataStorage Component. The Comet [16] component within the Mesh Server provides streaming real time data to the Web Browser through a secure SSL Internet connection. Comet builds on the HTTP protocol handshake and it ensures that the HTTP connection remains open for a long time between client and server for continuous data streaming. This enables us to provide a live Google Map display of the sensor data in the Web Browser, where the user can graphically select one or a group of sensors, check their status, receive real-time alerts, or select and modify the behavior of a group of sensors. The Web Browser uses JavaScript to communicate with Comet over the Internet. The Web Browser also communicates with the Web Portal. The Web Portal provides the Web Browser with access to historical data stored in the Database. Sensor selection queries from the Web Browser, which can be map-based or query-based, also request the sensor information from the database through the Web Portal. When the Web Browser receives the sensor information from the Web Portal, it then generates the request and sends it to the sensors through the Mesh Server to the Mesh Portals and eventually to the sensor nodes.

**Figure 7.**
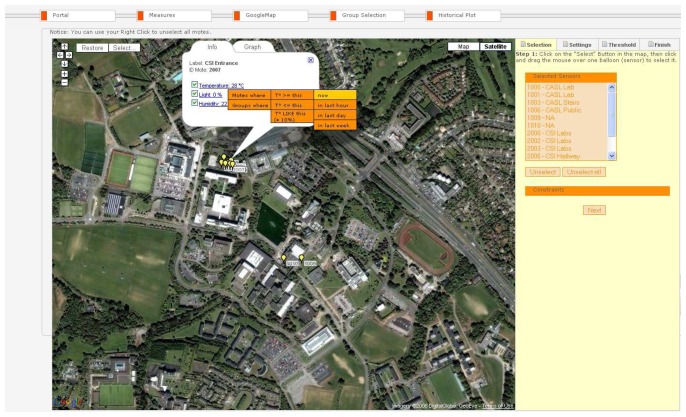
Map-based monitoring and node selection.

A central feature of the SUMAC Web interface is the map-based tool, shown in Figure 7, which leverages GoogleMap's open interface. This tool illustrates the sensor node locations on the satellite or street map. Obtaining the nodes' locations requires the collection of GPS coordinates of the sensors while or after deploying them. This can be done with a PDA and GPS receiver connected through a wireless Bluetooth interface. At each sensor node, a user click on the GPS receiver to get the GPS coordinates of the node. The coordinates are sent from the GPS receiver to the PDA through the wireless Bluetooth connection. The PDA then stores the GPS coordinates of the current node in an XML file that links each set of GPS coordinates to a unique sensor node ID. Repeating this step at every node ensures that the XML file contains the GPS coordinates of all the nodes. The XML file is then loaded into the Database, which can then provide the location of each node to the Web Portal.

Through the map-based tool, users can click on the balloons on the map, which represent the sensor nodes at the selected location. Clicking on specific sensor nodes in the map opens a callout box that displays the current sensor readings at that node. In Figure 7, the node with ID 2007 is selected. The callout that appears upon clicking on this node displays the current temperature, humidity and light readings at that location. Every time the server receives a new reading from this node, these reading are updated automatically in the callout on the map through the open Comet connection. Users can select any subset of these readings to view through the check boxes next to each reading. If a user is only interested in humidity data, the user can uncheck the boxes for temperature and light, which reduces the effective streaming load on the Comet connection. Clicking on the link for one of the metrics displays additional menus for automatically selecting nodes that belong to the same group or that share similar reading patterns as the selected node. In the example in Figure 7, the user graphically selects nodes or groups of nodes that have had higher temperature than node 2007 in the past hour. The IDs of the selected nodes then appear in the “Selected Sensors” box at right hand side of the screen.

The graph tab in the callout also allows the user to view the recent sensor reading plotted versus time. To select a group of sensors in the map tool, users can also click on the “Select” button at the upper left corner of the map. The user then clicks, holds, and drags their pointer to create a rectangle around the target group of nodes on the map. The selected nodes then change color to yellow, and the ID's of these nodes appear in the box on the right titled “Selected Sensors”. Buttons for unselecting one or all sensors are also available in this tab.

**Figure 8.**
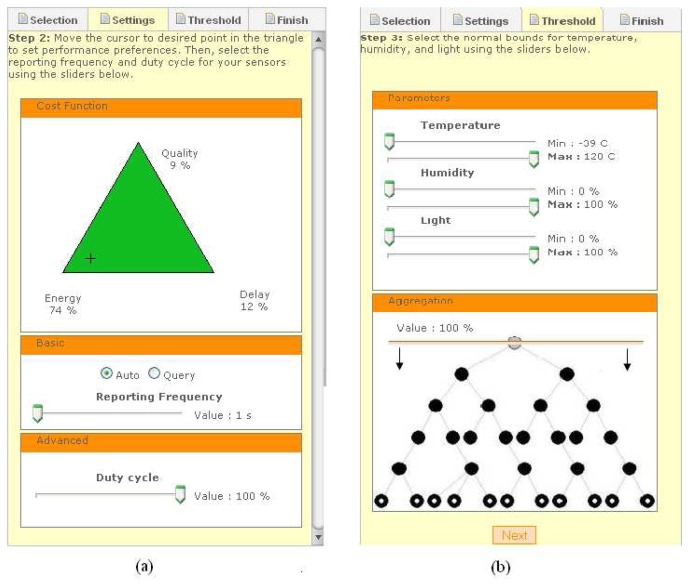
(a) performance settings; (b) threshold and reporting frequency settings.

Once a group of sensor nodes are selected, the user can set the performance metrics for the selected nodes, by clicking the “Settings” tab in the right box of the main GoogleMaps tab. The “Settings” tab is shown in Figure 8(a). The triangle tool within this tab allows the user to graphically set the relative importance of the three cost function metrics from equation 1 for tailoring the network performance to their application needs. By simply dragging the cursor closer to energy, the user indicates a higher preference for energy efficiency, as the example in Figure 8(a) shows. Note that the user can also set absolute performance metrics (not shown here), such as the desired system lifetime or maximum delay, which are then mapped by a background process to appropriate cost function metrics and aggregation policy. The user also sets the reporting frequency (sampling period) and the duty cycle through the sliders below the graphical triangle. Finally, the user can select whether to place the selected sensors in Auto (time-driven reporting) or Query (query-driven reporting) modes, before proceeding to the next step in the “Thresholds” tab, which is shown in Figure 8(b).

The “Threshold” tab enables the user to set maximum and minimum thresholds for each of the sensor readings. These settings are used by the sensors to detect anomalies in the monitored area. Whenever a sensor value exceeds its thresholds, the affected node stops aggregating data and wakes up its neighbors to report this potential event of interest (see Figure 4). The balloon corresponding to this node in the map also turns red to indicate this event to the user. The server also learns of this event and decides, based on the setting that the user had initially entered, how to adapt the aggregation level and cost function metrics in response to this event.

The “Threshold” tab also allows the user to visually verify and adjust the aggregation level in the network. By clicking and dragging the horizontal line up or down the network tree shown at the bottom right of Figure 8(b), the user can vary the portion of aggregated data in the network. Manually adjusting the aggregation value overrides the nominal aggregation value set automatically by the system based on the cost metrics selection in the triangle tool. For example, in Figure 8(b), the system has set the aggregation level to 100%, which means that all reading from within the selected group of nodes are aggregated and only one averaged reading arrives at the server. This is a consequence of assigning energy a high weight (74%) in the triangle tool at the earlier step (see Figure8(a)). If the user is not satisfied with this aggregation policy, the user can simply slide down the aggregation level to reduce the portion of aggregated data, bearing in mind that this reduces the weight for energy efficiency in the routing policy.

After selecting all these parameters, the user is prompted to confirm these settings, after which the server sends the corresponding configuration requests to the selected sensor nodes.

**Figure 9.**
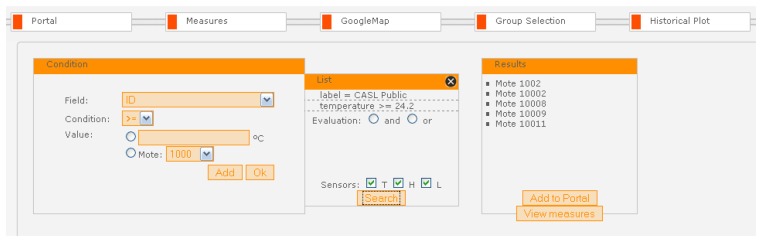
Sensor group selection interface.

The SUMAC Web portal also supports query-based selection of sensor groups through a powerful yet intuitive interface within the “Group Selection” tab, on the basis of hop count, data resolution, sensed values, GPS coordinates, and sensor tags, as shown in Figure 9. Through this interface, users can set the first rule by selecting a field to check, with the associated condition, and value. The user then clicks the “Add” button to include this rule in the query to the Database. Users can then add as many rules as required by repeating this process, and they can select “And” or “OR” to determine how the rules should be checked in the database. Once all the rules have been entered, clicking the “Search” button runs the composite query from the Web Browser to the Database through the Web Portal to get a list of all the nodes that satisfy these rules, which appears in the Group Selection tab as well. If the results are satisfactory, the user can issue a request to configure this group of nodes through the Google Maps tab, or add this list of nodes to the main Portal tab, so it will appear every time the user logs on.

In the example of Figure 9, the user has created a query to select sensors that are located in the public area of the CASL building, which is indicated by the label ‘CASL Public’, and whose temperature reading is greater than 24.2°*C*. Clicking the “Search” button generates a query to the Database through the Web Portal. The query results in a list of sensor node ID's to be sent back to the Web Browser and displayed in the “Results” box of the Group Selection tab. In the current example, the query returns three node ID's, which the user can add to the Web Portal or view the live sensor measurements.

**Figure 10.**
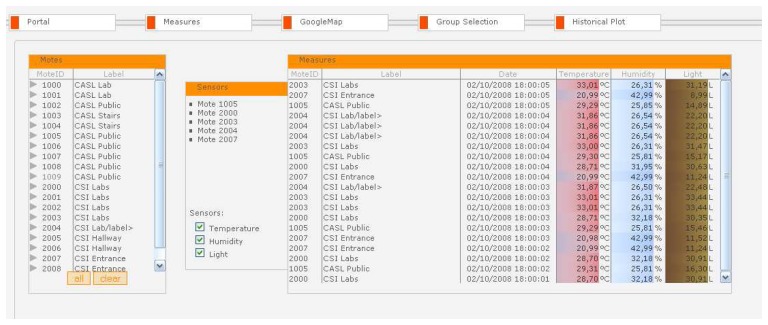
Real-time viewing of sensor data.

Real-time viewing of sensor data in the SUMAC Web portal is available by selecting a subset of nodes or a subset of sensors for selected nodes, either through the map-based tool, the group selection tool, or the “Measures” tab. Figure 10 shows a screenshot of the Measures feature. In this simple example, six motes are available for viewing. The user has selected three nodes, with ID's 10004, 10007, and 10018. Although these nodes include temperature, humidity, and light sensors, the user has opted to view only temperature and light values by selecting the corresponding check boxes in the sensors box. The selected sensor values from these 3 motes appear in the measures box on the right. The left-most column in the measures box includes the node ID. The second column includes the node label, which in this example groups nodes according to their physical location. The third column includes a timestamp of the reading. The next 2 columns contain the temperature and the light reading of the node respectively. Both these columns are color-coded. For temperature, warm values are shown in red, where the brighter the redness of the value, the warmer the temperature. The same applies for light, where different shades of brown indicate the level darkness, and brighter yellow shades indicates stronger light.

**Figure 11.**
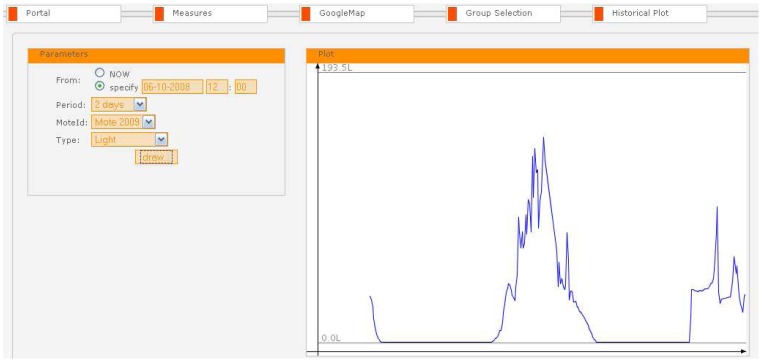
Historical plotting feature.

The historical plot tab in the SUMAC Web interface enables the user to view stored sensor samples from any past interval. The user can specify the desired starting time for the plot, the time window into the past for which to show the readings, and the sensor node of interest. Clicking the “Draw” button generates the plot. Figure 11 shows an example plot that was generated on October 6, 2008, for the last 48 hours in the entrance of UCD's CSI building, showing the light variation trend between night and day, as well as other short term luminance variations due to varying cloud cover outside in Dublin.

## 4. Performance Evaluation

This section focuses on the performance evaluation of SUMAC's adaptive fidelity mechanism. To evaluate the adaptive fidelity mechanism, we have performed empirical testbed experiments at our lab at UCD's Computer Science and Informatics Centre using a single cluster of 20 Tmote Sky nodes. The same network scenario was used for all the experiments. The software implementation uses nesC and leverages TinyOS version 2.

The 20 nodes are placed in an indoor area spanning two floors. Through TinyOS's low power listening feature, the duty cycle for all nodes is set to 30%. All nodes sample their sensors every 4 seconds and send their data towards the base station. Although our routing cost function from the CTM strategy supports energy and delay metrics, the experiments here use only ETX for better benchmarking by other TinyOS developers. Of course, the collected results would inform future experiments on setting cost function weights, mainly for managing the energy/delay tradeoff. Each test lasted for fifteen minutes. The network diameter varied between 5 to 6 hops, depending on interference conditions.

**Figure 12.**
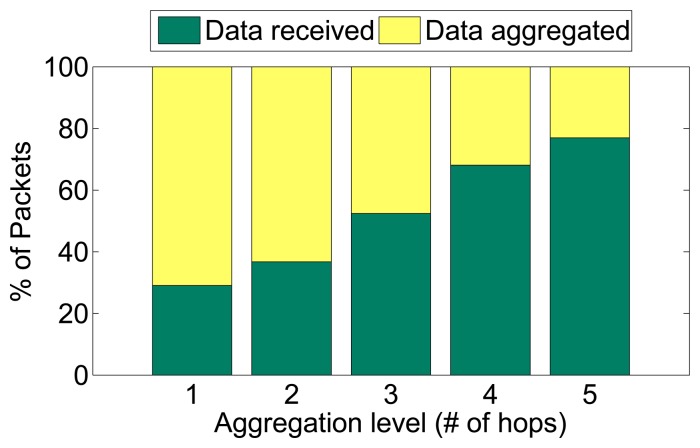
Percentage of packets transmitted and aggregated versus aggregation levels.

Figure 12 illustrates the traffic load effect of setting different aggregation levels, i.e. on the basis of hop count from the base station. The figure only shows aggregation levels up to 5 hops, as there were not always nodes at the sixth hop. When aggregation occurs at hop 5, 25% of the generated packets are aggregated, while 75% of them are received at the base station. As expected, setting a lower aggregation level causing a higher percentage of the packets to be aggregated, and a smaller percentage of them to be received at the base station. This trend is steady, and at an aggregation level of 1 hop, more than 70% of the generated packets are aggregated while only 30% are actually received at the base station. These values are obviously topology dependent, but they also confirm that the trend of traffic savings for lower aggregation levels.

**Figure 14.**
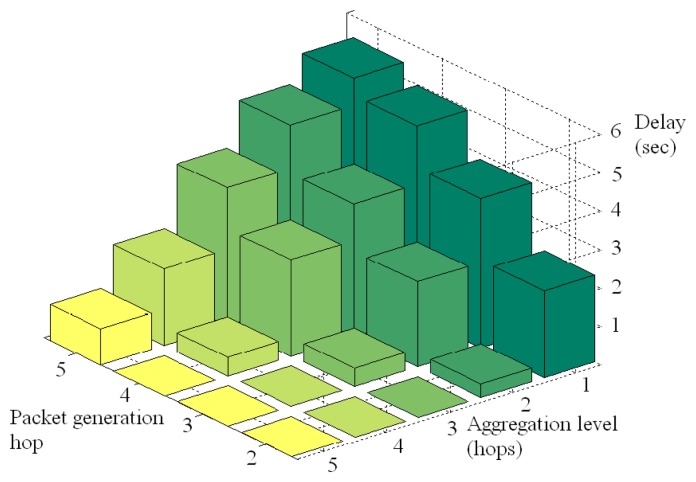
Observed delay for different aggregation levels and observation hops.

Figure 14(a) shows the resulting average number of radio transmissions to deliver a single packet from its source to the base station as the aggregation level varies. This metric is averaged over all the nodes in the network. For an aggregation level of 5, which is the closest case to having no aggregation, the average radio transmissions per packet is almost 3, which is equivalent to the median hop count in the network. This effect is intuitive as a node at hop 5 requires 5 transmissions to reach the base station with no aggregation, just like a node at hop 1 requires a single transmission. As the aggregation level moves closer to the base station, the average number of radio transmissions per packet decreases steadily to reach 1.2 transmissions at an aggregation level of 1 hop. In large networks with hundreds to thousands of nodes per cluster, the reduction in radio transmissions per packet can have substantial energy savings.

To put the traffic reduction effects in perspective, Figure 14(b) shows the averaged power consumption of nodes that are 1 hop away from the base station for different aggregation levels. These nodes, often referred to as critical nodes, actually determine the network lifetime since they must forward the packets of all other nodes, so their energy consumption is critical for the network lifetime. As the aggregation level moves closer to the base station, the power consumption of critical nodes decreases significantly. Recall from Figure 12 that an aggregation level at hop 5 leaves 75% of the packets unaffected by aggregation, while that value shrinks to 30% for an aggregation level of 1. This reduction in traffic load accounts for the reduction of power consumption of critical nodes by a factor of half when the aggregation level is shifted from hop 5 to hop 1. This effectively corresponds to doubling the network lifetime. In addition, Figure 14(b) also reveals that, in the specific topology of our experiment, the difference in power consumption of critical nodes for aggregation levels 2, 3, and 4 is less pronounced, though the trend of lower power consumption for a lower aggregation level holds. These results have an impact on the energy/delay tradeoff. For example, aggregating at level 2 or at level 3 does not change the power consumption of critical nodes much, but aggregating at level 3 may incur significantly less delay on aggregated packets than at level 2. The delay behavior of our aggregation is explored next.

The traffic and energy savings of our aggregation come at the cost of increased delay, which is il lustrated in Figure 14. This figure plots delay versus the aggregation level and the packet generation hop. The packet generation hop is the hop count of the node that generated the packet, while aggrega tion level is the hop count setting for aggregation. An aggregation level at hop count 1, which provided the highest traffic reduction, also yields the highest delay. In fact, packets generated at hop 5 have the highest delay, as they have to await the timer firing at each intermediate hop. As the aggregation level increases, the delay steadily decreases, while maintaining the trend that packets generated deeper in the network experience higher delay for the same aggregation level. We also note that the delay at the first hop after the aggregation level is not zero. For example, the delay for packets generated at hop 5 and an aggregation level at hop 5 yields almost a delay of 1 second. Similar situations occur for packets at hop 4 with aggregation level 4, and at lower hops as well. In fact, the plot exhibits symmetry for all delay for packets generated at one hop higher than the aggregation level, at two hops higher than the aggregation level, and so on, regardless of the aggregation level setting. These symmetries can be used to set guidelines for delay consequences of aggregating data, which can in turn be combined with user delay constraints to set cost function weights autonomously.

## 5. Discussion and Conclusion

This paper has presented the SUMAC system for large scale sensor networks that targets monitoring of geographically dispersed sensor clusters. The SUMAC architecture introduces a mesh network overlay to extend the range of sensor clusters in order to cover larger geographic areas and to maximize user ownership of the data path. The data from the mesh network then reaches the server, which supports both real-time data access, visualization, and network control as well as running historical queries and analysis. All components of the SUMAC architecture have been implemented and integrated, and the verification of the end-to-end performance, robustness and scalability of the system has revealed that the system performs well, and additional experiments are ongoing to validate this further.

SUMAC adopts an end-to-end approach to large scale monitoring applications, which includes the sensors, mesh, server planes. At the sensors plane, SUMAC introduces a suite of mechanisms for customization of the network performance through high level policies set by the user. These mechanisms include adaptive fidelity, which dynamically adapts the level of aggregation in the network to the current conditions, and a user-configurable cost function that determines routing paths in the network on the basis of application requirements. SUMAC also provides a feedback mechanism between adaptive fidelity and the cost function, to ensure that reconfiguration requests to the sensors jointly consider aggregation and routing topology, and adapt the network configuration autonomously to changing conditions without further user intervention.

The mesh network plane serves as a medium-range overlay for the sensors. Gateway nodes between the sensor and mesh plane merge several sensor packets into a single UDP packet and forward through the Mesh network until the packet reaches a Mesh Portal, which then forwards the packet to the Mesh Server in the server plane.

The SUMAC server design targets scalable data management for supporting tens of thousands of sensors nodes in a large number of clusters and deployments. The server provides live streaming data to the user through the Web browser, as described in Section 3.4., as well historical data storage and querying through a sharded, cloud-based relational database.

Work on SUMAC is ongoing to refine the feedback mechanism that autonomously manages the network setting according to high level policies set by the user. We are conducting extensive testbed experiments to establish the relationships between aggregation, the cost function, and data delivery to the user.

We have implemented and verified the functionality of all the server components for 2 clusters of 20 sensor nodes each located at the Computer Science and Informatics Centre and at the Complex and Adaptive Systems Laboratory. We are currently running extensive simulations on the server to test its scalability and delay constraints. To perform these simulations, we are using the Amazon Elastic Compute Cloud service (http://aws.amazon.com/ec2/) where we can simulate deployments of tens of thousands of nodes and the resulting delay behavior and resource usage of the server components. Our initial results indicate that a query to access historical data from the Web Browser for a network deployment of 1,000 nodes requires about 5 seconds delay on a Pentium 4 dedicated PC. Similar queries for a network of 10,000 nodes incurs a delay of about 5 minutes, and a network of 20,000 nodes results in a delay of about 20 minutes. We expect that our ongoing simulations will further reduce delay and reveal further trends which can be later generalized to data management and storage of large sensor deployments. Note that access to live real-time data streams is much shorter, in the order of a few seconds.

In addition, we are improving the front-end interface by adding features to graphically indicate to the user the current state of nodes on the map. For example, features under development include color coding of nodes in the map which are aggregating, and another color to indicate nodes whose neighbors have detected an event of interest. The improved interface will also provide more advanced grouping functionality for easier manipulation of node configuration. It will also include an additional advanced option in to enable users to specify maximum cluster sizes, so that the server can allocate addresses accordingly to each base station.

As a representative optimization mechanism developed within SUMAC, this paper has introduced the concept of adaptive fidelity by which users can set elaborate policies for specifying conditions for enabling aggregation and overriding it. The results in this paper have explored the energy/delay tradeoff and have determined that shifting the aggregation level from the 5th hop to the 1st hop from the base station can double the network lifetime at the cost of higher delay.

The results also provide initial guidelines for enabling the system to autonomously manage the energy/delay with aggregation. The user can set bounds and requirements through the graphical interface on energy and delay requirements, and the system could then determine the appropriate aggregation level, and the weights of the cost function of the CTM, in order to optimally configure the network for achieving the user-specified performance requirements.

## Figures and Tables

**Figure 6.**
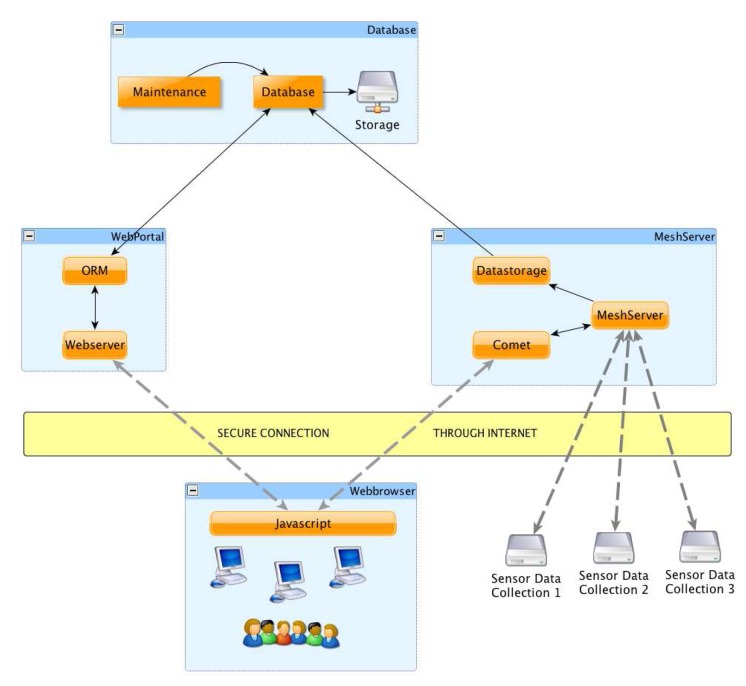
The SUMAC server architecture.

**Figure 13.**
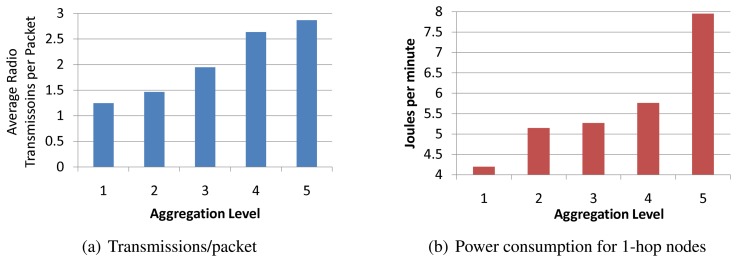
Traffic and Energy Savings at different aggregation levels.
